# The influence of interpersonal relationships on college students’ physical activity: chain-mediated effects of social support and exercise motivation

**DOI:** 10.3389/fpsyg.2025.1567122

**Published:** 2025-06-18

**Authors:** Yiqing Wang, Yue Wang, Moran Lv, Zhichen Cheng, YuLiu Tao

**Affiliations:** School of Physical Education, Soochow University, Suzhou, Jiangsu, China

**Keywords:** interpersonal relationships, physical activity, social support, exercise motivation, chain-mediated effects

## Abstract

**Background:**

While interpersonal relationships are known to benefit psychological health and personal development, their role in shaping physical activity behaviors among university students has yet to be thoroughly investigated. Gaining insight into the mechanisms linking these relationships to physical activity is crucial for developing effective strategies to encourage more active lifestyles in this demographic.

**Objective:**

This study seeks to investigate how interpersonal relationships affect physical activity in university students, with a particular focus on the sequential mediating roles of social support and motivation to exercise. The findings are intended to inform theory and guide the design of practical interventions to enhance physical activity among this population.

**Methods:**

A total of 635 university students were surveyed using a random sampling approach and a set of validated instruments, including the Comprehensive Diagnostic Scale for Interpersonal Relationships, the Physical Activity Motivation Scale, the Social Support Scale for Physical Activity, and the short version of the International Physical Activity Questionnaire (IPAQ-SF). Data analysis was conducted with SPSS 27.0, employing methods such as independent sample *t*-tests, one-way ANOVA, Pearson correlation analysis, Harman’s single-factor test to check for common method bias, and bootstrapped mediation testing using the PROCESS macro.

**Results:**

Interpersonal relationships were found to significantly influence university students’ physical activity, with this effect partially explained by the sequential mediation of social support and motivation to exercise. More specifically, interpersonal relationships were negatively associated with both social support and exercise motivation (*β* = −0.097, *p* = 0.014; *β* = −0.126, *p* = 0.001), whereas social support was positively associated with motivation to exercise (*β* = 0.316, *p* < 0.001). When all variables were included in the model, both social support and exercise motivation showed significant positive effects on physical activity levels (*β* = 0.241, *p* < 0.001; *β* = 0.127, *p* = 0.002). Mediation analysis further revealed a significant total indirect effect (value = −0.091) of social support and motivation to exercise on the relationship between interpersonal relationships and physical activity, with all three pathways—via social support, via motivation, and via the combined path of social support and motivation—yielding significant mediation effects.

**Conclusion:**

This study underscores the importance of interpersonal relationships, social support, and exercise motivation in shaping physical activity among university students, with particular emphasis on the interconnected mediation pathways among these factors. The results offer a theoretical basis for developing targeted physical activity interventions, highlighting the need to address these elements collectively rather than in isolation. Future studies are encouraged to explore how these relationships hold across diverse populations and cultural settings and to use longitudinal methods to establish causal links better.

## 1 Introduction

Insufficient physical activity has emerged as a major public health concern worldwide (Haseler and Haseler, 2022). It contributes to a range of chronic health conditions, including sarcopenia, metabolic syndrome, obesity, and hypertension (Booth et al., 2012; Eaton and Eaton, 2017). It can also lead to a variety of psychosocial issues, including diminished self-esteem and heightened levels of depression and anxiety (Happell et al., 2012). The growing prevalence of social media has led university students to spend more time on electronic devices like smartphones and computers, which in turn has increased sedentary behavior and substantially reduced their physical activity levels. Studies indicate that physical activity typically declines as individuals transition from late adolescence into early adulthood (Corder et al., 2019; Kjønniksen et al., 2008). A multinational survey covering university students in 23 countries revealed that nearly half do not engage in adequate physical activity, contributing to a deterioration in overall health (Pengpid et al., 2015). Regular physical activity has been widely recognized and utilized in both clinical and preventive health settings to help prevent and manage numerous chronic conditions (Dodge et al., 2024). It has been shown to reduce the risk of cardiovascular disease, enhance sleep quality, lower the likelihood of certain cancers, and even support better mental health (Crooke et al., 2020).

Physical activity (PA) refers to any movement of the body generated by skeletal muscles that require energy expenditure, which encompasses a wide range of activities, including recreational exercise, commuting between places, work-related tasks, and domestic chores (Caspersen et al., 1985). Physical activity is shaped by a complex interplay of personal, social, and environmental factors (Pedrelli et al., 2015). While most existing studies emphasize individual-level determinants, such as lifestyle habits, attitudes, perceived benefits, and intentions (Trost et al., 2002), and environmental aspects like facility access, weather, and safety (Humpel et al., 2002), less attention has been given to social variables beyond social support (Booth et al., 2000). Notably, the role of interpersonal relationships remains underexplored. From a social-psychological perspective, interpersonal relationships are emotional bonds formed through direct interactions with others. These relationships represent multidimensional support systems involving peers, teachers, and others, which can significantly influence both the motivation and opportunity for university students to participate in physical activity (Romaguera et al., 2011).

The social-ecological model suggests that various factors across personal, social, and environmental domains shape behavior. Among these, an individual’s motivation to exercise, their interpersonal connections, and the support they receive from others play pivotal roles in determining physical activity levels (Biddle et al., 2011; Romaguera et al., 2011). Despite this, few studies have examined how interpersonal relationships exert their influence. Thus, it is essential to explore whether such relationships impact physical activity indirectly—by fostering greater social support and enhancing exercise motivation.

Drawing on survey data from university students in Guizhou Province, China, this study explores whether interpersonal relationships influence students’ engagement in physical activity and whether this relationship is mediated, either independently or sequentially, by social support and motivation to exercise. Our findings indicate that stronger interpersonal ties may enhance physical activity through these underlying pathways. These insights deepen our understanding of the psychological and social mechanisms driving physical activity and carry meaningful implications for promoting physical and mental well-being in university populations.

## 2 Literature review and hypotheses

As a key element of social interaction, interpersonal relationships play an important role in shaping university students’ participation and persistence in physical activity. Kim and colleagues found that more physically active students tend to maintain healthier and more positive interpersonal ties (Kim et al., 2021). Effective communication and emotional exchange within relationships may foster a supportive environment that indirectly encourages greater involvement in exercise (Putnam et al., 1994; Costigan et al., 2019). However, findings in this area are not entirely consistent. For instance, Zhao’s study reported no direct effect of interpersonal relationships on physical activity but did point to the potential mediating role of factors such as motivation (Zhao et al., 2024). These mixed results indicate that the impact of interpersonal relationships may operate through complex psychological and behavioral mechanisms, highlighting the need for further investigation into how such relationships influence physical activity among university students.

In the framework of the social-ecological model, exercise motivation and social support independently contribute to promoting health-related behaviors. Social support refers to individuals’ perceptions and experiences of being loved, cared for, and valued, as well as the mutual aid and assistance they receive within their social networks (Sarason et al., 1983). As university students’ social and living environments continue to evolve rapidly (de la Sablonniere, 2017), the importance of social support for their physical and mental well-being has become increasingly pronounced. Among this population, such support typically stems from interpersonal and social connections. Numerous studies have found a positive association between social support and physical activity (Kirchhoff et al., 2008; Young and Stewart, 2006; Yasunaga et al., 2014). In a systematic review of 19 studies, Anne identified family members, peers, and teachers as key sources of social support influencing physical activity (Standiford, 2013). The interconnectedness of interpersonal relationships, social support, and physical activity in university students suggests that social support may mediate the relationship between interpersonal relationships and physical activity.

Exercise motivation may mediate the relationship between interpersonal relationships and physical activity in university students. Defined as the psychological impetus that drives individuals to begin and maintain regular exercise, motivation is widely recognized as a key determinant of physical activity behavior (Standage et al., 2008). Exercise motivation is a pivotal factor in promoting participation in physical activity (Baranowski et al., 1998). Based on Self-Determination Theory (SDT), motivation is typically divided into intrinsic and extrinsic types. Intrinsic motivation arises when individuals engage in physical activity out of genuine interest or enjoyment rather than due to external incentives or pressures. Jacobs observed that intrinsic motivation plays a crucial role in helping individuals form consistent and sustainable exercise habits (Deci and Ryan, 2000). Supporting this, Rich et al. found that motivation is a significant predictor of health behaviors in general (Rich et al., 2015). Motivation to exercise is directly and indirectly associated with physical activity through mechanisms such as outcome expectations, perceived barriers, and self-regulatory behavior (Ayotte et al., 2010). Among university students, increased motivation has been shown to enhance leisure satisfaction and psychological resilience, which in turn boosts their willingness to engage in physical activity (Yu et al., 2024). Furthermore, individuals with strong interpersonal relationships tend to receive greater social support—both of which are key elements influencing physical activity, with motivation being central to Self-Determination Theory. These findings collectively suggest that both exercise motivation and social support play important roles in promoting physical activity among university students.

Interpersonal relationships have been shown to influence physical activity among university students directly and indirectly through social support and exercise motivation. While prior research has identified numerous factors affecting physical activity, few studies have examined these four variables—interpersonal relationships, social support, motivation to exercise, and physical activity—within a unified framework. In particular, the mediating roles of social support and motivation remain insufficiently tested. Furthermore, the pathways through which interpersonal relationships affect student physical activity are still not well understood. This study seeks to address this gap by focusing on university students, clarifying how interpersonal relationships shape their physical activity behaviors, and offering theoretical and practical contributions to promoting student health. Based on this goal, we propose the following hypotheses:

*H1*: Interpersonal relationships significantly influence physical activity levels in university students.

*H2*: Social support mediates the impact of interpersonal relationships on physical activity among university students.

*H3*: Exercise motivation mediates the link between interpersonal relationships and physical activity in university students.

*H4*: Exercise motivation and social support function as sequential mediators in the relationship between interpersonal relationships and physical activity among university students.

## 3 Research methodology

### 3.1 Objects of study

A simple random sampling method without replacement was adopted to survey university students from several institutions in Guizhou Province, China. Using official student ID lists provided by the universities, participants were randomly selected through a computer-generated number sequence, ensuring that every student had an equal chance of selection and no individual was chosen more than once. This method effectively eliminated human bias in the sampling process, thereby enhancing the representativeness and objectivity of the sample.

Out of 719 questionnaires collected, the research team applied strict quality control measures to ensure data reliability. Questionnaires with more than 10% missing responses or completed in an unusually short time were excluded. After removing responses with substantial errors or missing key information, a total of 635 valid questionnaires were retained, resulting in an effective response rate of 88.3%. The final sample consisted of 331 male (52.1%) and 304 female (47.9%) students, with an average age of 21.2 years (SD = 1.1), ranging from 20 to 23 years. Participants represented a range of academic backgrounds, including humanities (27.2%), sciences (20.8%), engineering (51.8%), and arts and sports, indicating academic diversity. Geographically, students were sampled from various universities across both urban and rural regions of Guizhou Province, reflecting a broad institutional and demographic distribution. Table 1 provides a detailed summary of participants’ demographic characteristics, including gender, age, academic year, ethnicity, residence, and field of study.

**Table 1:** Demographic characteristics of participants (*N* = 635).

Demography variant	Total (*n* = 635) Male *n* = 331 (52.1) Female *n* = 304 (47.9)				
	Mean ± SD or *n* (%)			*x*^2^/t	*p*
(a person’s) age	21.2 ± 1.1	21.2 ± 1.1	21.1 ± 1.1	1.34	0.178
Han ethnic group	313 (49.3)	169 (51.1)	144 (47.4)	0.863	0.353
National minority	322 (50.7)	162 (48.9)	160 (52.6)		
Municipalities	92 (14.5)	39 (11.8)	53 (17.4)	5.220	0.074
Townships	418 (65.8)	230 (69.5)	188 (61.8)		
Countryside	125 (19.7)	62 (18.7)	63 (20.7)		
Liberal arts	173 (27.2)	14 (4.2)	159 (52.3)	210.643	<0.001
Science and engineering as academic subjects	132 (20.8)	66 (19.9)	66 (21.7)		
Engineering class	329 (51.8)	250 (75.5)	79 (26.0)		
Art and physical education	1 (0.2)	1 (0.3)	0 (0.0)		
First-year university student	3 (0.5)	3 (0.9)	0 (0.0)	11.431	0.010
Second-year university student	275 (43.3)	161 (48.6)	114 (37.5)		
Third-year university student	350 (55.1)	164 (49.5)	186 (61.2)		
Fourth-year university student	7 (1.1)	3 (0.9)	4 (1.3)		

Although local regulations do not require formal ethical approval, this study followed international ethical requirements to ensure the rights and well-being of participants. To ensure that the many subjects who participated in the survey were willing participants in this study, the research team set up an informed consent form on the first page of the questionnaire. All participants were informed of the purpose of the study and the procedures and that they had the right to withdraw at any time without consequence. The study also assured participants that their responses would be anonymous and used only for academic research. These measures were consistent with the ethical principles of confidentiality, informed consent, and voluntary participation.

### 3.2 Research tools

#### 3.2.1 Interpersonal relations

To assess interpersonal difficulties among participants, this study utilized the Comprehensive Diagnostic Scale of Interpersonal Relationships developed by Zheng Richang. The instrument includes 28 items equally distributed across four dimensions: verbal communication, social engagement, interpersonal interaction, and cross-gender relations. Each item is answered with “Yes” or “No,” with “Yes” responses assigned 1 point and “No” assigned 0, yielding a total score range from 0 to 28. Subscale scores are calculated by summing the items within each category. Higher scores reflect a greater degree of interpersonal difficulty in that area. In this study, the scale demonstrated strong reliability, with a Cronbach’s alpha of 0.917.

#### 3.2.2 Motivation to exercise

The Chinese version of the Motives for Physical Activity Measure – Revised (MPAM-R) was used in this study to assess participants’ exercise motivation. Originally developed by Ryan and Deci (2000) within the framework of Self-Determination Theory, the scale was adapted into Chinese by Chen Shanping and colleagues. It comprises five motivational dimensions: enjoyment, competence, appearance, health, and social interaction. Participants responded to each item on a 5-point Likert scale (1 = “Not at all true” to 5 = “Very true”), with higher total scores reflecting stronger overall exercise motivation. The scale demonstrated excellent reliability in the current study, with a Cronbach’s alpha of 0.942.

#### 3.2.3 Social support for physical activity

To measure perceived social support for physical activity, this study used a 12-item scale developed by Cao Yunxia and colleagues for Chinese university students (Cao et al., 2024). The instrument utilizes a 5-point Likert scale, assessing support across three domains: family, peers, and others. Participants indicated their agreement with each statement on a scale from 1 (strongly disagree) to 5 (strongly agree), with higher scores reflecting higher levels of perceived social support. In the current study, the scale demonstrated strong reliability, with a Cronbach’s alpha of 0.908.

#### 3.2.4 Physical activity

To assess university students’ physical activity levels, this study employed the widely used International Physical Activity Questionnaire - Short Form (IPAQ-SF) (Hagströmer et al., 2006). The questionnaire consists of seven items that evaluate physical activity patterns over the past 7 days, including time and frequency spent walking, performing moderate-and vigorous-intensity activities, and sedentary behavior. Based on IPAQ scoring guidelines, metabolic equivalent (MET) values were assigned to each activity type: 3.3 for walking, 4.0 for moderate-intensity activity, and 8.0 for vigorous-intensity activity. Weekly MET scores were calculated using the formula: MET-minutes/week = minutes per day × days per week × corresponding MET value. The total MET score was obtained by summing all activity-specific MET scores.

Data were processed in accordance with IPAQ’s official data cleaning recommendations, which included truncating and removing implausible values. Specifically, entries were excluded if any daily activity exceeded 960 min, weekly time for any activity exceeded 1,260 min (21 h), total MET score exceeded 20,000 MET-min/week, or activity frequency was reported as more than 7 days per week. These procedures helped ensure the validity of the physical activity data. Based on the IPAQ classification system, participants were then categorized into low, moderate, or high activity level groups, which served as the basis for evaluating physical activity status.

### 3.3 Data analysis

Descriptive and correlational analyses were conducted using SPSS 27.0 to examine the relationships among interpersonal relationships, social support, exercise motivation, and physical activity in university students. Structural equation modeling was performed in AMOS 22.0 to evaluate the proposed mediation model, and mediation effects were tested using the PROCESS macro. Model fit was assessed using standard indices (χ^2^/df = 2.729, CFI = 0.926, RMSEA = 0.068), indicating acceptable model adequacy and supporting the reliability of the mediation framework. This analytical approach enabled a more detailed understanding of how interpersonal relationships, social support, and motivation contribute to physical activity behaviors. The mediation analysis results provided insights into the presence and strength of indirect effects, enhancing the interpretation of the interrelationships among key variables.

## 4 Results and analysis

### 4.1 Common method bias test

In order to cautiously assess possible common method bias, the Harman one-way common method bias test was used in this study. An unrotated exploratory factor analysis was conducted on all entries, including the variables of interpersonal relationships, motivation to exercise, social support, and physical activity. The results showed that the coefficient of variation of the largest eigenvalue was 17.35%, which was lower than the critical value of 40% (Podsakoff et al., 2003); initial analyses suggest that common method bias is not a serious concern in this study. Nevertheless, it is important to acknowledge the limitations of Harman’s single-factor test. Although widely used for its simplicity, this method lacks sensitivity to minor or latent sources of bias and may not effectively differentiate between true construct variance and systematic measurement error. Future studies should consider adopting more rigorous techniques to evaluate common method variance.

### 4.2 Descriptive statistics and correlation analysis

To explore the associations among the core variables, this study performed descriptive and Pearson correlation analyses on interpersonal relationship distress, exercise motivation, social support, and physical activity. Detailed results are reported in Tables 2, 3.

**Table 2:** Descriptive statistics and reliability coefficients (*N* = 635).

Variant	Total (*n* = 635) Male *n* = 331 (52.1) Female *n* = 304 (47.9)			
	Mean ± SD			Cronbach’s alpha
Interpersonal relationship	0.27 (0.24)	1.9 (1.7)	1.9 (1.6)	0.917
Social aspect	0.31 (0.28)	0.31 (0.29)	0.31 (0.28)	
Conversation	0.35 (0.31)	0.32 (0.31)	0.38 (0.31)	
Heterosexual intercourse	0.26 (0.26)	0.27 (0.26)	0.25 (0.24)	
The way one treats people	0.17 (0.23)	0.19 (0.25)	0.15 (0.20)	
Social security (pensions, medical insurance)	3.61 (1.03)	3.68 (1.04)	3.53 (1.01)	0.916
Emotional support	3.84 (1.37)	3.66 (1.28)	4.03 (1.43)	
Evaluative support	3.65 (1.15)	3.79 (1.09)	3.50 (1.18)	
Informative support	3.66 (1.13)	3.78 (1.15)	3.52 (1.08)	
Instrumental support	3.35 (1.26)	3.54 (1.22)	3.15 (1.27)	
Motivation for exercise	3.57 (0.70)	3.53 (0.69)	3.60 (0.73)	0.942
Health motivation	3.65 (0.79)	3.60 (0.79)	3.71 (0.80)	
Appearance (linguistics)	3.59 (0.78)	3.53 (0.74)	3.66 (0.81)	
Pleasure motive	3.58 (0.75)	3.55 (0.71)	3.61 (0.80)	
Social motivation	3.48 (0.80)	3.47 (0.77)	3.50 (0.82)	
Motivation for competence	3.54 (0.78)	3.51 (0.74)	3.56 (0.82)	

**Table 3:** Correlation analysis of interpersonal relationships, physical activity, social support and motivation to exercise.

Variant	1	2	3	4
1. Physical activity	1			
2. Motivation to exercise	0.204**	1		
3. Interpersonal relations	−0.042	−0.153**	1	
4. Social support	0.291**	0.330**	−0.094*	1

**p < 0.01, *p < 0.05.

Table 2 summarizes the descriptive statistics for the three primary constructs: interpersonal relationships, social support, and exercise motivation. The internal consistency of the scales used in this study was strong, with Cronbach’s alpha coefficients ranging from 0.917 to 0.942, indicating reliable measurement across all variables.

As indicated in Table 3, there was a significant positive correlation between physical activity and exercise motivation (*r* = 0.204**, *p* < 0.01), suggesting that individuals who are more motivated to exercise are also more likely to engage in physical activity—supporting the Self-Determination Theory’s assumption of alignment between motivation and behavior. Similarly, social support showed a positive correlation with physical activity (*r* = 0.291**, *p* < 0.01), indicating that individuals who receive more support from family, peers, or others tend to be more physically active. This is consistent with existing literature highlighting the facilitative role of social support in health behavior adoption. Additionally, social support was positively correlated with exercise motivation (*r* = 0.330**, *p* < 0.01), suggesting that social support may play both a direct role in influencing physical activity and an indirect role by enhancing internal motivation. Finally, interpersonal distress was found to be negatively correlated with exercise motivation (*r* = −0.153, *p* < 0.01), indicating that individuals who experience more difficulties in interpersonal relationships may be less likely to feel motivated to exercise—possibly due to reduced social engagement or the impact of negative affect.

### 4.3 Tests for mediating effects

To investigate the underlying mechanism linking interpersonal relationships and college students’ physical activity, we conducted a mediation analysis using structural equation modeling while controlling for gender, age, and academic year. As shown in Table 4, interpersonal relationship distress was found to negatively predict both social support and exercise motivation (*β* = −0.126, *p* = 0.001, *R*^2^ = 0.134), lending empirical support to Hypothesis 1. Moreover, social support was positively associated with exercise motivation (*β* = 0.316, *p* < 0.001). When all three variables—interpersonal relationships, social support, and exercise motivation—were included in the model, both social support and exercise motivation emerged as significant positive predictors of physical activity (*β* = 0.241, *p* < 0.001; *β* = 0.127, *p* = 0.002; *R*^2^ = 0.107), thus supporting Hypotheses H2 and H3.

**Table 4:** Regression analysis between variables.

Regression equation (*N* = 635)		Fitness index			Significance of the coefficient	
Outcome variable	Predictor variable	*R*	*R^2^*	*F(df)*	*β*	*t*
Social security (pensions, medical insurance)	Distinguishing between the sexes	0.169	0.028	4.614	−0.075	−1.892
	(a person’s) age				−0.103	−2.438*
	Grade				−0.034	−0.804
	Interpersonal relationship				−0.097	−2.466
Exercise motivation	Distinguishing between the sexes	0.366	0.134	19.448	0.079	2.094*
	(a person’s) age				−0.032	−0.809
	Grade				−0.050	−1.244
	Interpersonal relationship				−0.126	−3.365
	Social security (pensions, medical insurance)				0.316	8.401
Physical activity	Distinguishing between the sexes	0.327	0.107	12.556	−0.084	−2.175
	(a person’s) Age				0.002	0.054
	Grade				−0.037	−0.911
	Interpersonal relationship				−0.002	−0.053
	Social security (pensions, medical insurance)				0.241	5.964
	Motivation for exercise				0.127	3.129*

***p < 0.001, **p < 0.05, and *p < 0.1; All regression models control gender, age, and grade level.

### 4.4 Chain mediation effect test

We employed the Bootstrap method via the PROCESS macro to examine the mediating effects. The analysis revealed that both social support and exercise motivation significantly mediated the relationship between interpersonal relationships and physical activity, with a total indirect effect of −0.091. Three distinct mediation pathways were identified:

(1) Interpersonal relationships → social support → physical activity: This pathway showed a significant indirect effect of −0.049, with a 95% confidence interval from −0.103 to −0.005.

(2) Interpersonal relationships → exercise motivation → physical activity: The mediation effect for this pathway was −0.033, with a 95% CI of −0.070 to 0.008, indicating significance.

(3) Interpersonal relationships → social support → exercise motivation → physical activity: This sequential pathway also reached significance, with an indirect effect of −0.008 and a 95% CI from −0.020 to 0.000.

These findings underscore the pivotal role of social support and exercise motivation as mediators, enhancing our understanding of how interpersonal dynamics influence physical activity among college students. Detailed results are presented in Table 5.

**Table 5:** Mediating effects of social support for physical activity and exercise motivation between interpersonal relationships and physical activity.

Trails	Efficiency value	Boot standard error	95% CI lower limit	95% CI limit
Interpersonal relationships → social support	−0.421	0.171	−0.757	−0, 086
Social support → exercise motivation	0.219	0.026	0.168	0.270
Interpersonal relationships → exercise motivation	−0.377	0.112	−0.597	−0.157
Social support → physical activity	0.116	0.019	0.078	0.155
exercise motivation → physical activity	0.089	0.028	0.033	0.144
Total indirect effect	−0.091	0.033	−0.158	−0.030
Interpersonal relationships → social support → physical activity	−0.049	0.025	−0.103	−0.005
Interpersonal relationships → exercise motivation → physical activity	−0.033	0.016	−0.070	−0.008
Interpersonal relationships → social support → exercise motivation → physical activity	−0.008	0.005	−0.020	0.000

## 5 Discussion

Difficulties in interpersonal relationships may hinder college students from participating in physical activity. Research has indicated that strong social ties—with family members, friends, or romantic partners—can positively influence individuals by providing behavioral role models, thereby promoting healthier habits and increasing physical activity levels (Shaffer et al., 2024). Meanwhile, research has also shown that effective interpersonal communication can stimulate students’ interest in physical activity and boost their motivation to participate (Pop, 2014). Consequently, strained interpersonal relationships may undermine an individual’s psychological well-being, which in turn reduces their enthusiasm and motivation to engage in physical activity.

Interpersonal relationships affect college students’ physical activity levels by shaping the degree of social support and exercise motivation. Specifically, when students encounter difficulties in their social interactions—with family, peers, or classmates—they may experience heightened loneliness and feel less seen or validated by others. This diminished social support weakens their motivation to be physically active, ultimately leading to lower levels of physical activity (Carter et al., 2022; Belanger and Patrick, 2018). Secondly, interpersonal conflicts can trigger psychological problems such as social anxiety in college students, which in turn diminishes their willingness to exercise and negatively impacts their overall physical activity levels. Prior research has shown that intrinsic motivation plays a key role in fostering greater involvement in physical activity (Cox et al., 2008; Di Domenico and Ryan, 2017); this observation is consistent with the Self-Determination Theory, which emphasizes that an individual’s motivation is a crucial driver of behavior (Deci and Ryan, 2000). Thus, healthy interpersonal relationships can effectively encourage greater physical activity among college students, partly by enhancing social support and strengthening exercise motivation, which act as mediators in this association.

Interpersonal difficulties may indirectly predict physical activity levels through a sequential pathway involving diminished social support and reduced exercise motivation. In essence, when college students struggle with social relationships, they often experience a decline in perceived social support. This isolation undermines their motivation to engage in physical activity, eventually leading to lower participation. As Self-Determination Theory outlines unmet relational needs can prompt students to socially withdraw, limiting their interactions with peers and classmates. Such withdrawal weakens both instrumental support—such as having someone to exercise with—and emotional support, like receiving encouragement to stay physically active. When college students are surrounded by low social support, their expectations for the benefits of physical activity often decline, resulting in reduced participation.

## 6 Conclusion

Physical activity plays a crucial role in enhancing college students’ overall health and reducing the risk of chronic diseases. As such, it is essential to explore the underlying patterns and mechanisms that drive physical activity participation. Researchers have increasingly focused on factors at the individual and environmental levels, along with the influence of social support at the broader societal level (Pedrelli et al., 2015; Trost et al., 2002; Humpel et al., 2002; Booth et al., 2000; Romaguera et al., 2011; Kim et al., 2021; Putnam et al., 1994). Given that individuals are fundamentally shaped by their social relationships and cannot exist in isolation from their social environment, this study examined how interpersonal relationships influence physical activity and shed light on the mechanisms underlying this connection.

This study yielded three key findings. First, interpersonal difficulties among college students were found to hinder engagement in physical activity, whereas strong and healthy relationships were associated with higher levels of participation. Second, both social support and exercise motivation independently mediated the link between interpersonal relationships and physical activity. Third, a sequential mediation pathway was identified: supportive relationships foster greater perceived social support, which in turn enhances motivation to exercise, ultimately promoting increased physical activity.

Our primary contribution empirically validates how interpersonal relationships shape social support, motivational processes, and physical activity behavior. This not only deepens the understanding of the role interpersonal factors play in health-related behaviors but also uncovers the underlying mechanisms through which social relationships can be leveraged to improve health outcomes. These findings offer valuable insights for designing targeted interventions to enhance physical activity and well-being among college students.

This study has several limitations. First, our sample consisted solely of college students whose unique lifestyles, psychological characteristics, and social contexts may limit the generalizability of the findings to other age groups or social populations. Future research should replicate and expand these findings in more diverse demographic groups. Second, the use of cross-sectional data restricts our ability to draw causal inferences between variables. Longitudinal designs in future studies would be valuable for uncovering the directional and temporal dynamics of these relationships.

## Data Availability

The original contributions presented in the study are included in the article/Supplementary material, further inquiries can be directed to the corresponding author/s.
