# Supplementary material for: The influence of interpersonal relationships on college students’ physical activity: chain-mediated effects of social support and exercise motivation
Source: Front Psychol. 2025 Jun 18;16:1567122. doi: 10.3389/fpsyg.2025.1567122 (PMC12213819; doi:10.3389/fpsyg.2025.1567122)
Supplement: Supplementary file 1 [file Supplementary_file_1.docx]

Supplementary Material

# Supplementary Figures and Tables

## Supplementary Figures


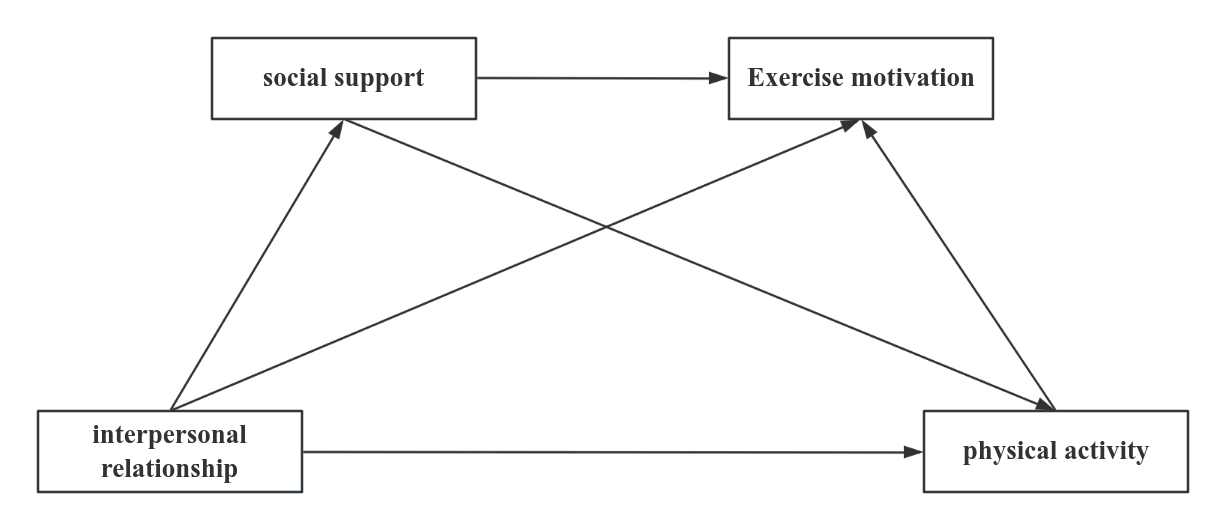


**Figure 1 Hypothetical model of the effect of interpersonal relationships on physical activity of university students**

**
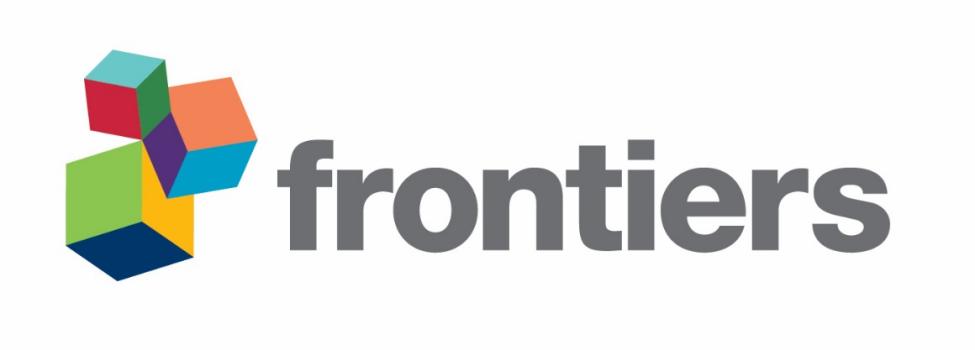
**
